# Structural properties of native and conjugated black soldier fly (*Hermetia illucens*) larvae protein via Maillard reaction and classification by SIMCA

**DOI:** 10.1016/j.heliyon.2021.e07242

**Published:** 2021-06-08

**Authors:** Vusi Vincent Mshayisa, Jessy Van Wyk, Bongisiwe Zozo, Silvio D. Rodríguez

**Affiliations:** a Department of Food Science and Technology, Cape Peninsula University of Technology, Bellville, 7535, South Africa; b Department of Chemistry, Cape Peninsula University of Technology, Bellville, 7535, South Africa; c Instituto de Biodiversidad y Biología Experimental y Aplicada (IBBEA), CONICET –Facultad de Ciencias Exactas y Naturales, Universidad de Buenos Aires, Buenos Aires, Argentina

**Keywords:** Chemometric method, Conjugation, Maillard reaction, Soft independent modelling of class analogy, Principal component analysis, Glycation

## Abstract

Black soldier fly (*Hermetia illucens*) has received considerable interest as an alternative protein source. Aqueous solutions of black soldier fly larvae (BSFL) protein and glucose (2:1 w.w^−1^, pH 9) were heated at 50, 70 and 90 °C, for 2–10 h at 2 h intervals, respectively. The zeta-potential (ζ) of BSFL-Glu conjugates heat-treated at 70 °C ranged from -10.25 to -25.25 mV while the native BSFL protein ranged from -12.84 to -16.70 mV. The ζ-potential analysis revealed that the glycation reaction modified the surface charge density of the BSFL protein as a function of reaction time and temperature. In addition, an increase in thermal stability of the BSFL-Glu conjugates was observed by means of Thermo-gravimetric analysis (TGA) and differential scanning calorimetry (DSC). Fourier transform infrared spectroscopy (FT-IR) analysis indicated that the most apparent structural changes in the BSFL protein were in the amide I and amide II region. Well-separated clusters permitting differentiation between native BSFL and BSFL-Glu conjugates were observed by using principal component analysis (PCA) on FT-IR spectra. At 50, 70 and 90 °C the first two principal components (PC1 and PC2) showed an accumulated total variance of 91, 96 and 95%, respectively. A classification efficiency of 91% was obtained when using soft independent modelling of class analogy (SIMCA). Infrared spectroscopy combined with SIMCA is a powerful tool to monitor the formation of edible insect protein–sugar conjugates by Maillard reaction. As a result, combining FT-IR spectroscopy with multivariate techniques (PCA and SIMCA) exhibited a strong potential to differentiate between native and glycated protein samples from black soldier fly larvae.

## 1 Introduction

The food industry is exploring alternative protein sources for human consumption in response to global concerns about food security and protein malnutrition due to the growing population. Novel protein sources such as algae, pea, rapeseed, duckweed and insects have received a great deal of research interest in the past decade (Boland et al., 2013; Van-der Spiegel et al., 2013; Kim et al., 2016; Leni et al., 2019). Edible insects such as crickets (*Acheta domesticus*), mealworm (*Tenebrio molitor*), Mopani worm (*Imbrasia. Belina*) and black soldier fly larvae (*Hermetia illucens*) have emerged as promising alternatives since they have a high fat and protein content, high feed conversion ratio, lower environmental footprint and higher economic value (Wang and Shelomi, 2017; Gould and Wolf, 2018; Huang et al., 2018). In particular, proteins extracted from black soldier fly larvae and used as novel food ingredients in different food applications might have greater success in terms of consumer acceptance since some consumers show aversion towards consuming whole insects (Patel et al., 2019).

To further promote the application of black soldier fly larvae (BSFL) proteins in the food industry, it is imperative to understand the structural and thermal properties which influence their techno-functional behaviour such as solubility, emulsification, oil and water binding capacity and gelation in food systems. In order to enhance the techno-functionality of proteins, food manufacturers attempt by all means to avoid chemical agents that may be potentially toxic and hazardous to consumers (Chevalier et al., 2001; Medrano et al., 2009; Corzo-Martínez et al., 2017).

Therefore, conjugation via the Maillard reaction has been deemed as a generally regarded as safe (GRAS) process since it occurs spontaneously and does not involve extraneous chemicals. During food processing and storage, the Maillard glycation between amino groups of proteins and carbonyl groups of reducing sugars is inevitable. The initial stage of this reaction includes the condensation of the carbonyl group with the available ε-amino group, resulting in the development of Amadori reaction products by forming a Schiff base with the release of water (O'Mahony et al., 2017; Pirestani et al., 2018). The reaction is greatly accelerated by heat and can be induced under both wet and dry states. However, the latter is not feasible from an industrial point of view since it requires extended reaction times up to several days or weeks. Moreover, factors such as reaction pH, temperature and time can influence the physico-chemical and techno-functional properties of the formed conjugates. These conjugates are diverse, complex, not fully understood and thus assessment of the reaction with a single analysis is not effective.

Studies have shown that FT-IR (Fourier transform infrared spectroscopy) can be used to evaluate the structural properties of proteins induced by conjugation in wet or dry state (Oliver et al., 2009; Wang et al., 2013; Mellado-Carretero et al., 2019). FT- IR is a useful analytical technique for rapid, sensitive, low-cost, reliable, precise and non-destructive analysis of chemical compounds in food matrices and provides an alternative to wet-chemical and laborious methods (Rodríguez et al., 2019). It is considered as a green analytical technique since it eliminates the use of hazardous chemicals. To further enhance the use of FT-IR and apply a multi-pronged approach, it is often used in amalgamation with multivariate statistical methods known as chemometrics. Chemometric methods such as Partial least squares discriminant analysis (PLS-DA), Principal component analysis (PCA), Cluster analysis (CA) or Soft independent class modelling analogy (SIMCA) allows scientists to elicit vital information from big sets of data (Balan et al., 2020). Mellado-Carretero et al. (2019) used pairwise SIMCA models to show significant chemical differences in protein-polysaccharide conjugates. SIMCA showed 100% classification efficiency when applied to detect adulteration of milk samples with formalin. In addition, Oliver et al. (2009) reported that FT-IR was able to classify between non-glycated and glycated sodium caseinate conjugates when PCA and SIMCA were applied. To the best of our knowledge, there is a paucity of information on the application of FT-IR with chemometric methods on the conjugation of BSFL protein via the Maillard reaction and the knowledge underpinning insect protein-sugar interactions is still rather limited.

The aim of this study was to characterise the native BSFL protein and BSFL-Glu conjugates using novel analytical techniques, attenuated total reflection (ATR) FT-IR coupled with chemometrics (PCA and SIMCA). The complementary analysis techniques, thermal gravimetric analysis (TGA), differential scanning calorimetry (DSC), zeta potential and scanning electron microscopy (SEM) were also performed to further probe the structural modifications to the native BSFL protein and BSFL-Glu conjugates due to the Maillard reaction.

## 2 Materials and methods

### 2.1 Chemicals

All chemicals used were of analytical grade and were purchased from either Sigma-Aldrich (Aston Manor, South Africa) or Science World (Cape Town, South Africa) if not stated otherwise. Black soldier fly larvae (BSFL) was obtained from AgriProtein SA (Cape Town, South Africa).

### 2.2 Synthesis of Maillard conjugates, pH and browning index

BSFL-Glu conjugates were prepared according to the method of Mshayisa and Van Wyk and (2021) and Mshayisa (2016) with slight modifications. BSFL protein concentrate and glucose (2:1 w.w^−1^) were dispersed in 100 mL of 0.1 M phosphate buffer at pH 9. The mixture was transferred into 250 mL Schott bottles and heated at 50, 70 and 90 °C in a water bath for 0, 2, 4, 6, 8 and 10 h, respectively. After the heating period had elapsed, the resulting BSFL-Glu conjugates were immediately cooled in an ice bath. The native (as is or non-glycated) BSFL protein was also heated at the same temperature and time intervals as described above. In addition, unheated BSFL-Glu conjugates served as a control. The samples were freeze-dried and stored in air-tight screw-capped glass bottles at -80 °C until analysis.

### 2.3 Zeta potential surface charge (Zeta potential)

The ζ-Potential of native BSFL protein and BSFL-Glu conjugates of protein concentrates were determined using a Zetasizer Nano Series (Malvern Instruments, Malvern, Worcestershire, UK) as described by Ladjal-Ettoumi et al. (2016). Samples were diluted to 1% (w.v^−1^) protein concentration with Milli-Q water before measurement. Data was collected over at least five sequential readings and processed using the Smoluchowski mode (Schwenzfeier et al., 2013).

### 2.4 Scanning electron microscopy (SEM)

The surface morphology of the freeze-dried samples of the native BSFL protein and BSFL-Glu conjugates was examined using scanning electron microscopy (TM-3000, Hitachi Corporation, Tokyo, Japan). The sample was placed onto double-sided carbon adhesive tape attached to the specimen stubs. The surface structure of the sample was observed at 320 X magnification and in secondary electron mode at 15.0 kV.

### 2.5 Thermal analysis

The thermal properties that include differential scanning calorimetry (DSC) and thermo-gravimetric analysis (TGA) were evaluated on the freeze-dried heat-treated native BSFL protein and BSFL-Glu conjugates. In both, these analyses, approximately 4.0–6.0 mg of sample was weighed into an aluminium pan. A sealed empty pan was used as a reference. The DSC profile of the samples was evaluated using a thermal analyser (Q2000, Perkin Elmer, Singapore). The pans were sealed and heated from 30 to 180 °C at a heating rate of 20 °*C min*^−1^. The thermal stability behaviour of native BSFL protein and BSFL-Glu conjugates was evaluated using a TGA system (TGA-7, Perkin Elmer, Singapore). The analysis was performed from 30 to 650 °C at a heating rate of 20 °*C min*^−1^ with nitrogen as carrier gas at a flow rate of 50 mL min^−1^.

### 2.6 FT-IR spectra acquisition

All heat-treated native BSFL protein and BSFL-Glu conjugate samples in powder form were analysed using a Fourier transform infrared spectroscopy (FT-IR) equipped with a universal attenuated total reflectance (UATR) polarization accessory for spectrums (Spectrum 400, Perkin Elmer, Singapore). Prior to data collection of each sample, a background spectrum was collected and then the sample powders obtained by grinding in a mortar were placed directly covering the surface of the ATR crystal. All spectra were acquired by co-addition of 32 scans at a resolution of 4 cm^−1^ in the range of 400–4000 cm^−1^. The UATR crystal was cleaned with acetone to remove any residual contribution of the previous samples. To ensure reproducibility, spectra of each sample were acquired in triplicate.

#### 2.6.1 Chemometric approach

All the acquired sample spectra were analysed using the Unscrambler X software version 11 (CAMO Software, Oslo, Norway) (CAMO analytics – www.camo.com). The samples were separated into two groups for SIMCA: a training set (60 samples) and a prediction set (30 samples). All acquired spectrum was baseline corrected, transformed into absorbance units and normalized. Characteristic fingerprinting regions were established by exploring several peaks in the plotted raw spectra.

#### 2.6.2 Sample classification using SIMCA

SIMCA is a supervised PCA-based classification method in which each class is modelled independently and is applied to predict the class membership of the test set at a 5 % significance level. Primarily, a global model was developed. In the next step, class models for heated BSFL protein and BSFL-Glu conjugates based on the heating temperature and time were developed using a calibration data set (60 samples) and a test or prediction set (30 samples). The best fit-test set samples were classified to one of the developed class models. The statistical significance level for the SIMCA classification was set at 5%. This assumed that there was a 5% risk that a particular test sample would fall outside the class, even if it belonged to the class; while 95% of the test samples which truly belonged to the class would fall inside the class. The performance criterion is assessed by comparing each sample's identified class to its genuine class membership. By analyzing the specified class of each sample with its true class membership, the output criterion is determined. True positive identification (TP) refers to samples of a given class that are properly identified by the respective class, while samples are correctly rejected by the respective class model in true negative identification (TN). False negative identifications are defined as samples that belong to a given class but have been misclassified by the respective class. In false positive identifications, samples do not belong to a given class but were incorrectly recognized by the established class model. The evaluation parameters which determine the classification performance of each model are sensitivity, precision and specificity. Sensitivity (Eq. (1)) signifies the percentage of samples recognized by the model class in the prediction range, while specificity (Eq. (2)) represents the percentage of samples from other groups that the model class correctly rejects. The geometrical mean of precision and sensitivity (Eq. (3)) is essentially accuracy (Balan et al., 2020). Efficiency can be between 0 (sensitivity or specificity values are zero) and 1 (sensitivity or specificity values are zero) (sensitivity or specificity values are 1). Interpretations of the SIMCA class model were based on inter-class distance, the power of discrimination and class projections.

(1) $$\text{Sensitivity}=\frac{\text{TP}}{\text{TP}+\text{FN}}$$

(2) $$\text{Specificity }=\frac{\text{TN}}{\text{TN}+\text{FP}}$$

(3) $$\text{Precision}=\sqrt{\frac{\text{TP }\times \text{TN }}{\left(\text{TP}+\text{FN}\right)\times \left(\text{TN}+\text{FP}\right)}}$$

### 2.7 Data analysis

All data were subjected to multivariate analysis of variance (MANOVA) to ascertain whether the main effects resulted in significant differences in response variables. Duncan's multiple comparison *post hoc* test was used to test significant differences (p < 0.05) between individual means. SPSS 27.0 for Windows® was used for the statistical analyses and the level of confidence required for significance was selected at p < 0.05. The Unscrambler software, version 11 (CAMO Software, Oslo, Norway) was used to perform PCA and SIMCA. Graphs and figures were generated using Origin software, version 9.60 (Origin labs, Northampton, MA, USA) and The Unscrambler software.

## 3 Results and discussion

### 3.1 Zeta potential

**Figure 1:**
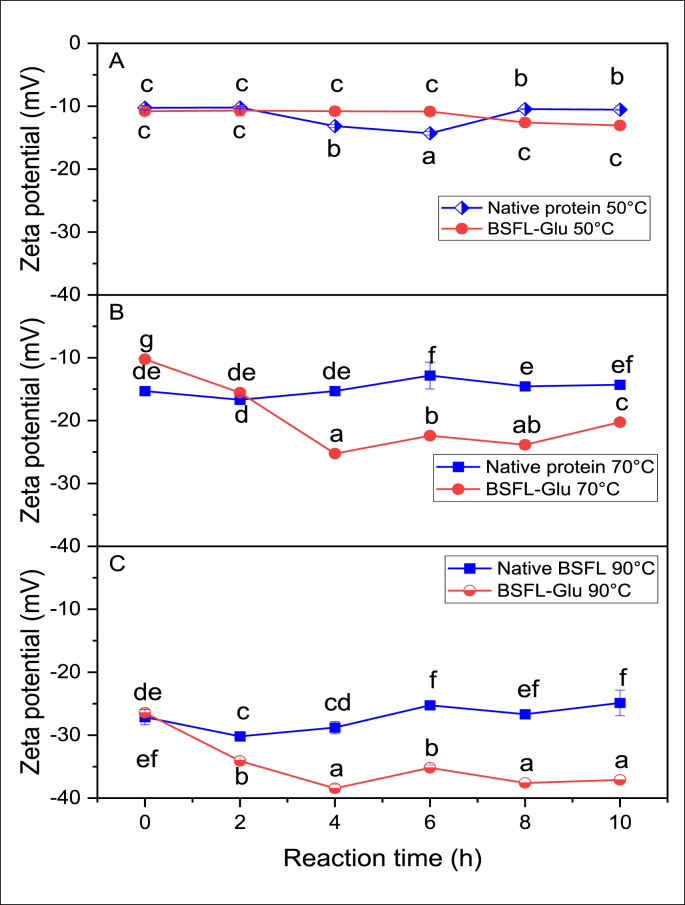
Zeta potential of native BSFL protein and BSFL-Glu conjugates heated at A) 50 °C, B) 70 °C and C) 90 °C as a function of time. Comparisons were carried out between values of the same series and values without the same letter(s) indicate a significant difference at p < 0.05.

Protein molecules in most cases carry a charge and this plays a significant role in interaction with other components in food matrices. The zeta potential (ζ) of proteins is a vital analysis that can be applied to optimise food product formulations for novel ingredients, predict interactions with surfaces and prediction of long-term stability. The ζ-potential of the native BSFL protein and BSFL-Glu conjugates heated at 50, 70 and 90 °C, respectively, is illustrated in Figure 1 A – C. Since most colloidal dispersions in aqueous media carries an electric charge, a negative ζ-potential indicates that the particles in suspension carry an overall negative charge. The ζ-potential of native BSFL protein heated at 50 °C ranged from -10.25 to -10.55 mV while BSFL-Glu conjugates ranged from -10.80 to -13.05 mV (Figure 1A). The BSFL conjugates heated at 70 and 90 °C exhibited lower ζ-potentials compared to the native BSFL proteins at the same heating temperature (Figure 1 B and C). This is might be due to conjugation with glucose which altered the surface charge distribution of BSFL protein, making more negatively charged groups such as –COOH and –OH more accessible due to the heat treatment. Moreover, the ζ-potential of BSFL-Glu conjugates prepared at 90 °C decreased from -27.15 to -37.11 mV as a function of reaction time. The linkages of the glucose to the BSFL protein during conjugation might lead to electrostatic screening and modifications of the surface charge of BSFL protein and thereby contributes to the observed lower ζ-potential. These results are in agreement with previous studies on pea protein isolate-maltodextrin conjugates. Proteins with a Zeta-potential higher than 30 mV absolute value are commonly known to be stable and do not flocculate (Mellado-Carretero et al., 2019). Generally, the ζ-potential of all samples remained negative at all reaction times tested. The covalent bond formed between the amine group of proteins and the carbonyl compounds of reducing sugars during the Maillard reaction results in a shift in the isoelectric point (pI) of proteins to a lower pH value. Therefore, the differences in the ζ-potential of the native BSFL protein and BSFL-Glu conjugates can be attributed to the structural modifications of the proteins induced by the heat treatment and to the unfolding of the protein leading to the exposure of more negative charges on the surface of the BSFL protein i.e. more stable to flocculation. Therefore, conjugated BSFL protein has the potential to be used in dressings and beverages which requires stability against flocculation.

### 3.2 Thermo-gravimetric analysis (TGA)

**Figure 2:**
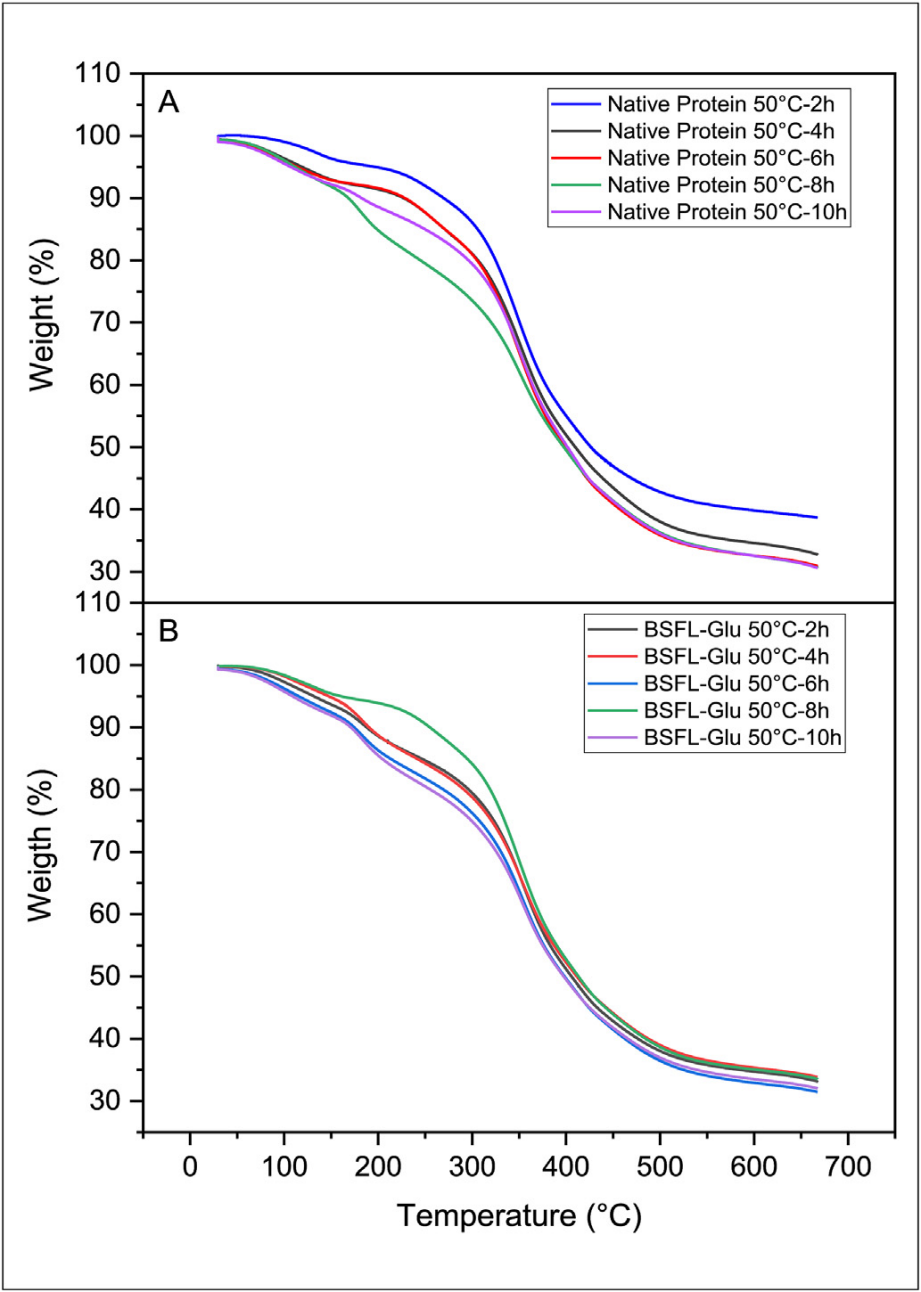
TGA scans of A) native BSFL protein and B) BSFL-Glu conjugates heated at 50 °C.

**Figure 3:**
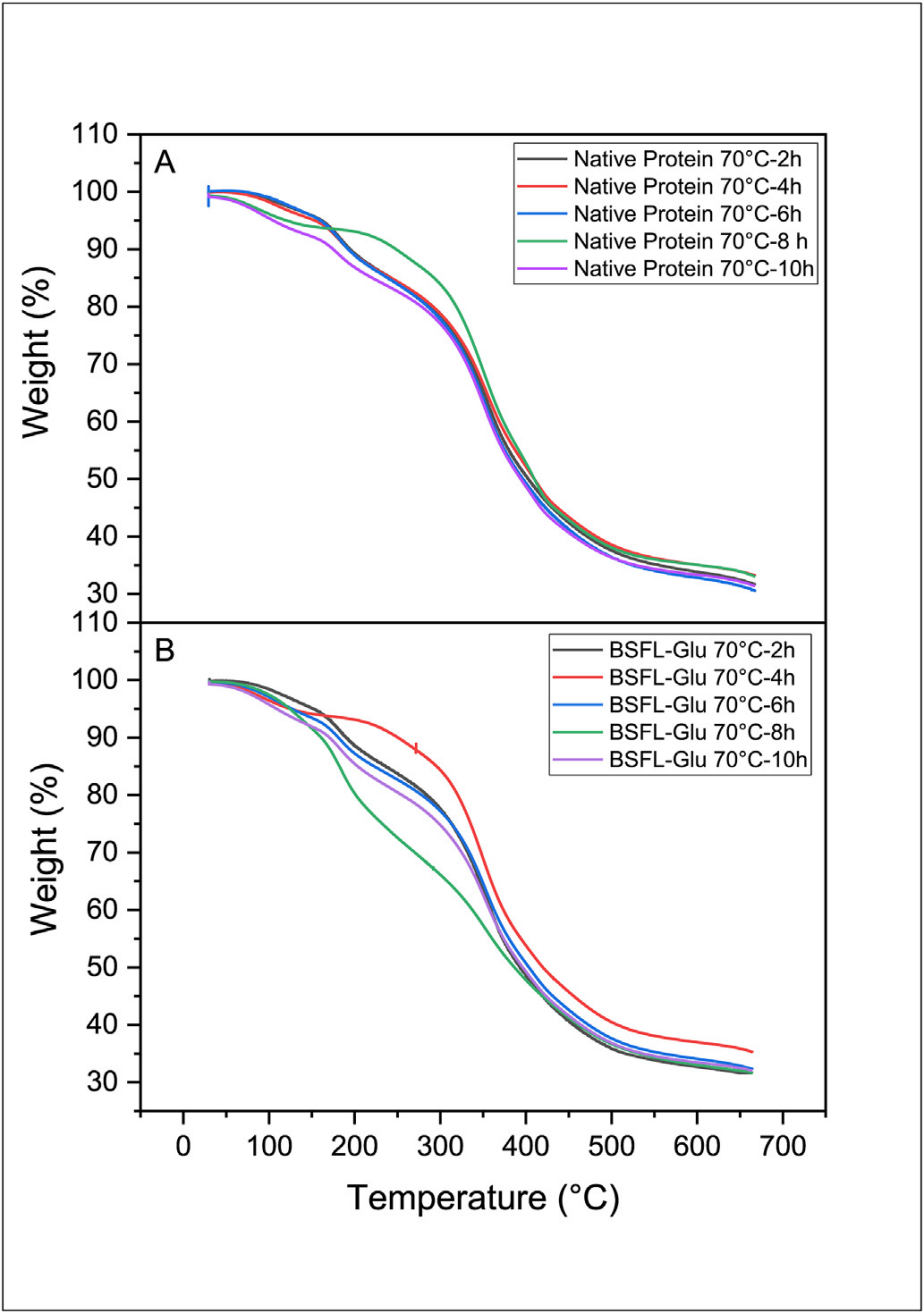
TGA scans of A) native BSFL protein and B) BSFL-Glu conjugates heated at 70 °C.

**Figure 4:**
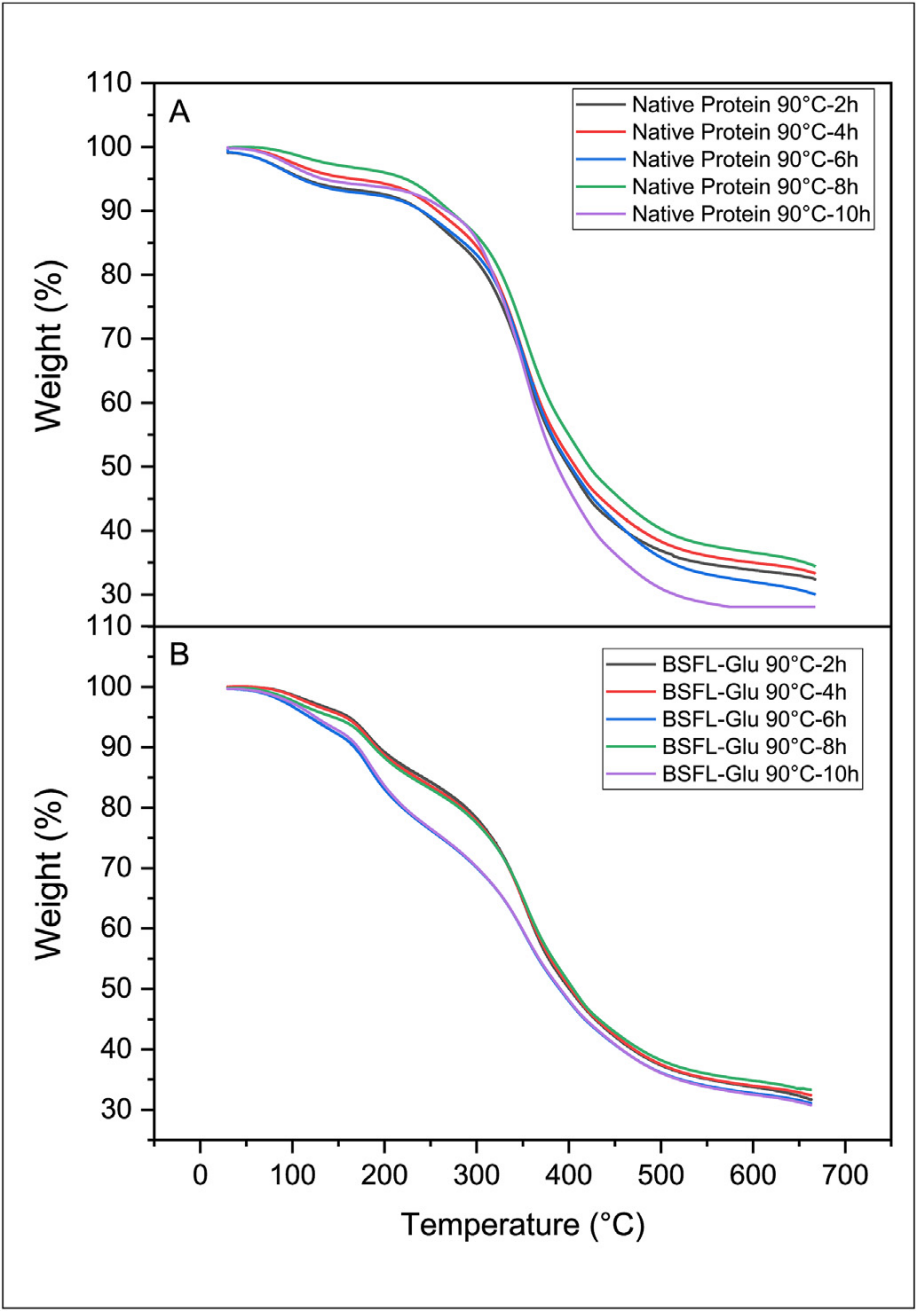
TGA scans of A) native BSFL protein and B) BSFL-Glu conjugates heated at 90 °C.

TGA is an analytical technique that has been widely used to monitor the mass (weight) of a substance as a function of temperature as the sample is subjected to a controlled environment and temperature increase program. The thermal stability of native BSFL protein and BSFL-Glu conjugates synthesised by heating at 50, 70 and 90 °C, for 2–10 h was assessed employing TGA in a temperature range between 30 and 650 °C. The TGA results of native BSFL protein and BSFL-Glu conjugates are shown in Figures 2, 3, and 4. By selecting the reaction temperature of 90 °C as a representative, it can be seen that the native BSFL protein undergoes three stages of thermal decomposition. The first stage of decomposition was attributed to the evaporation of free and loosely bound water and other volatiles in the temperature range 60–150 °C (Figure 4A). The second stage of the weight loss displayed by the heated native BSFL protein was the decomposition or degradation at approximately 150–300 °C. All the curves in this region are closer together signifying that the rate of decomposition is similar. The third stage from 450 °C–550 °C may be associated with the polypeptide decomposition in the native BSFL protein. It was observed that native protein heated for 10 h had the lowest residual mass after the heating process. In terms of the BSFL-Glu conjugates prepared at 90 °C (Figure 4B), the TGA thermograms revealed that the BSFL-Glu conjugates had approximately 10% weight loss due to moisture loss in the temperature range 0–150 °C.

BSFL-Glu conjugates prepared at 90 °C (6 h and 10 h) started to decompose at about 150 °C–210 °C and its weight loss sharply increased in the temperature range 250–500 °C (Figure 4A – B). The sharp decrease in the weight is due to the decomposition of conjugation or glycation products and the pyrolysis of other compounds. Compared with the native BSFL protein, the BSFL-Glu conjugates TGA thermograms exhibited a superimposed pattern in terms of the samples (6 h and 10 h) between 100 – 400 °C which were further away from the rest of the samples (Figure 4). Conjugates at 6 and 10 h had the most gradual slope of the weight versus temperature plot. The remaining products at this heating range (500–650 °C) could be other pyrolysis residues and ash (Figure 4B). This implies that these conjugates (6 h and 10 h) exhibit greater thermal stability compared to the other samples. The enhanced thermal stability of the BSFL-Glu can be ascribed to crosslinking and Maillard reaction between BSFL protein and glucose. The addition of glucose to the native BSFL protein via the Maillard reaction resulted in structural modifications in the BSFL protein, becoming more unfolded and this was observed by the SEM results described latter in section 3.7.

### 3.3 Differential scanning calorimetry (DSC)

After glycation, the tertiary/quaternary structure of food protein also changes, resulting in the functional properties being altered. It is vital to investigate such structural modifications since they are closely related to the functional properties of the protein in food. DSC can directly quantify heat changes in the sample during monitored temperature increases or decreases and can provide very important information about the sample's thermal stability (Liu et al., 2012).

The changes in thermodynamic parameters of heat-treated native BSFL protein and BSFL- Glu conjugates are presented in Table 1. Critical information on heat-induced protein unfolding and protein thermal stability is given by T_on_ and T_p_, respectively. The changes in enthalpy (ΔH) correspond to the energy requirement for transforming the ordered structure of proteins from native compact architectures to the unfolded state. The present results indicate that the thermal denaturation peak temperature (T_p_) for the heat-treated native BSFL protein at 50 °C ranged from 72.23 – 73.81 °C, with no significant differences (p > 0.05) between heating times (2, 4, 6, 8 and 10 h). On the contrary, at the same heating temperature (50 °C) BSFL-Glu conjugates exhibited significantly (p < 0.05) higher T_p_ in the range 78.37 and 78.92 °C.

**Table 1:** Thermal properties of heat-treated native BSFL protein and BSFL-Glu conjugates.

Test parameters	T_on_ (°C)	T_p_ (°C)	T_c_ (°C)	ΔH (J/g)
**Heat treatment at 50°C**				
Native BSFL 50 °C_2h	35.86 ± 0.40^e^	72.23 ± 0.04^a^	142.06 ± 0.00^e^	117.15 ± 0.04 ^de^
Native BSFL 50 °C_4h	34.65 ± 0.22^d^	73.02 ± 0.75 ^ab^	144.45 ± 0.71^f^	116.54 ± 0.04^d^
Native BSFL 50 °C_6h	34.62 ± 0.18^d^	73.06 ± 0.04 ^ab^	139.23 ± 0.64^d^	117.67 ± 0.75 ^de^
Native BSFL 50 °C_8h	38.94 ± 0.18^g^	73.92 ± 0.64^b^	136.78 ± 0.21^c^	118.07 ± 0.04 ^de^
Native BSFL 50 °C_10h	37.24 ± 0.00^f^	73.81 ± 0.01^b^	137.04 ± 0.71^c^	119.48 ± 0.75 ^de^
BSFL-Glu 50 °C_2h	32.06 ± 0.00^a^	78.63 ± 0.35^a^	129.31 ± 0.00^a^	113.26 ± 0.71^c^
BSFL-Glu 50 °C_4h	33.03 ± 0.00^b^	78.70 ± 0.24^c^	131.08 ± 0.00^b^	112.84 ± 2.83^c^
BSFL-Glu 50 °C_6h	32.07 ± 0.00^a^	78.92 ± 0.02^c^	129.92 ± 0.00^a^	110.12 ± 0.71^b^
BSFL-Glu 50 °C_8h	33.05 ± 0.00^b^	78.37 ± 0.45^c^	139.74 ± 0.00^d^	108.80 ± 0.35^b^
BSFL-Glu 50 °C_10h	33.66 ± 0.00^c^	78.71 ± 0.92^c^	143.73 ± 0.00^f^	106.25 ± 0.28^a^
**Heat treatment at 70°C**				
Native BSFL 70 °C_2h	36.80 ± 0.04^a^	74.76 ± 0.28^b^	128.41 ± 0.00^e^	124.52 ± 0.71^ef^
Native BSFL 70 °C_4h	35.06 ± 0.04^a^	74.52 ± 0.04^ab^	102.78 ± 0.00^a^	124.99 ± 0.04^f^
Native BSFL 70 °C_6h	36.82 ± 0.04^a^	74.36 ± 0.38^ab^	125.63 ± 0.00^d^	122.04 ± 1.41^e^
Native BSFL 70 °C_8h	41.43 ± 0.04^a^	73.92 ± 0.64^a^	130.30 ± 0.00^f^	122.07 ± 0.59^e^
Native BSFL 70 °C_10h	53.11 ± 0.27^a^	74.31 ± 0.69^ab^	125.54 ± 0.00^bc^	118.15 ± 1.07^d^
BSFL-Glu 70 °C_2h	35.09 ± 0.00^a^	81.62 ± 0.00^c^	102.75 ± 0.04^a^	74.30 ± 0.04^a^
BSFL-Glu 70 °C_4h	41.43 ± 0.04^a^	86.97 ± 0.01^d^	130.27 ± 0.04^f^	102.27 ± 0.31^c^
BSFL-Glu 70 °C_6h	33.99 ± 0.04^a^	88.28 ± 0.00^e^	125.51 ± 0.04^b^	98.87 ± 0.04^b^
BSFL-Glu 70 °C_8h	36.83 ± 0.00^a^	82.16 ± 0.00^c^	128.38 ± 0.04^e^	76.33 ± 2.83^a^
BSFL-Glu 70 °C_10h	36.82 ± 0.04^a^	86.84 ± 0.00^d^	125.60 ± 0.04 ^cd^	101.01 ± 0.04 ^bc^
**Heat treatment at 90°C**				
Native BSFL 90 °C_2h	30.47 ± 0.23^a^	75.26 ± 0.42^a^	126.42 ± 0.42^d^	105.43 ± 0.71^f^
Native BSFL 90 °C_4h	31.63 ± 0.14^c^	75.02 ± 0.75^a^	124.11 ± 0.15^b^	111.43 ± 0.42^g^
Native BSFL 90 °C_6h	31.25 ± 0.00^b^	74.86 ± 1.09^a^	125.61 ± 0.53^c^	117.70 ± 0.28^h^
Native BSFL 90 °C_8h	32.72 ± 0.00^d^	74.42 ± 1.34^a^	125.59 ± 0.40^c^	131.47 ± 0.64^j^
Native BSFL 90 °C_10h	33.47 ± 0.00^f^	74.31 ± 0.69^a^	133.34 ± 0.29^e^	128.22 ± 0.03^i^
BSFL-Glu 90 °C_2h	40.96 ± 0.00^j^	90.17 ± 0.05^b^	126.39 ± 0.07^d^	96.77 ± 0.01^d^
BSFL-Glu 90 °C_4h	33.06 ± 0.00^e^	90.37 ± 0.01^b^	124.43 ± 0.04^b^	97.68 ± 0.37^e^
BSFL-Glu 90 °C_6h	37.47 ± 0.00^h^	90.46 ± 0.01^b^	132.79 ± 0.29^e^	95.37 ± 0.35^c^
BSFL-Glu 90 °C_8h	40.75 ± 0.00^i^	90.57 ± 0.03^b^	77.89 ± 0.00^a^	71.29 ± 0.00^a^
BSFL-Glu 90 °C_10h	36.18 ± 0.00^g^	90.73 ± 0.05^b^	136.10 ± 0.00^f^	92.52 ± 0.01^b^

Onset temperature (T_on_), denaturation peak temperature (T_p_), conclusion temperature (T_c_) Enthalpy (ΔH). Values with different superscript in the same column for same heat treatment indicate significant differences (p < 0.05). Values reported as mean values (n = 2) and standard deviation.

Furthermore, the enthalpy (ΔH) of BSFL-Glu conjugates significantly decreased (p < 0.05) with increasing duration of heating. After the conjugation reaction, the decreased magnitude of the enthalpy (ΔH) can be attributed to the disturbance of the intramolecular forces of the BSFL protein when it is covalently bound to glucose. In this study, the steric spacers between the protein molecules can also be decreased by glucose molecules, which would facilitate aggregation by increasing the interactions of the surface hydrophobic binding sites. At 70 °C, no significant differences (p > 0.05) were observed in the onset temperature (T_o_) between the native BSFL protein and BSFL-Glu conjugates. Similar findings were reported by Tang et al. (2011) who showed that the T_p_ of glycated kidney bean vicilin (phaseolin) with glucose was significantly higher compared to the native samples.

Generally, at 90 °C the T_p_ for the BSFL-Glu conjugates ranged from 90.17 – 90.73 °C and was significantly higher (p < 0.05) compared to the heat-treated native BSFL protein (74.31–75.25 °C). These results suggest a remarkable increase in thermal stability of the BSFL-Glu conjugates which might be due to the unfolding of the protein structure which was initially ordered. Moreover, the denaturation temperature T_p_ of the conjugates increased with an increase in heat treatment (90 > 70 > 50 °C) which implied that the conjugates interfere with the process of native protein denaturation thus enhancing the thermal stability of BSFL-Glu conjugates. Throughout the food processing process, heat treatment may result in irreversible protein modifications due to the destruction of hydrogen and hydrophobic bonds or bridges, electrostatic and disulfide bonds interactions that stabilize the native conformations. The high thermostability of BSFL-Glu conjugates via the Maillard reaction (MR) under controlled conditions could make them good functional ingredients in heat-treated food products and expand their applications.

### 3.4 FTIR-ATR spectral analysis

**Figure 5:**
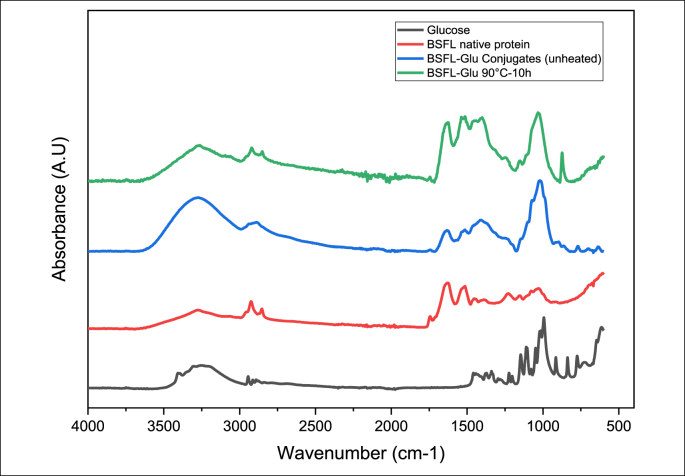
Attenuated total reflectance Fourier transform mid-infrared spectroscopy of glucose, native BSFL protein, BSFL-Glu conjugates (unheated) and BSFL-Glu 90 °C–10 h.

The spectroscopic investigation of polymeric particles, including protein, is complex due to the atomic vibrations emerging from various molecules. FT-IR spectroscopy is a useful non-destructive, precise and consistent analytical tool that can be utilised to probe structural modifications in protein-sugar systems since there are several readily identifiable regions of the mid-infrared spectrum where the chemical fingerprints of carbohydrates and proteins do not overlap significantly. The FT-IR spectra of glucose (unheated), native BSFL, BSFL-Glu (unheated, control sample) and BSFL conjugates (90 °C, 10 h) are depicted in Figure 5. The glucose spectrum reveals all normal features of glucose as previously described (Oliver et al., 2009). The strong absorption (C–O stretching) in the range 900–1100 cm^−1^ with a maximum at 1028 cm^−1^ is typical for carbohydrates. As can be seen in Figure 5, the spectra for the native BSFL protein reveals all the normal features of a protein spectrum with amide I and II in the 1624 and 1513 cm^−1^ region, respectively. These bands are due to stretching vibrations of the peptide linkages (C–N and C=O). The spectra of the conjugates (unheated and heated at 90 °C, 10 h) show that when the glucose was added, the IR band associated with carbohydrates (1028 cm^−1^, C–O stretching) was modified and increased in the resulting conjugates. Distinct conformational changes that occur as a result of interactions between the BSFL and glucose at a molecular or structural level are evident through the use of FT-IR. The BSFL-Glu conjugates heated at 90 °C for 10 h clearly shows an increased intensity in the amide I region (1624 cm^−1^). Moreover, the carbohydrate region 1022 cm^−1^ had an increased intensity in the conjugates compared to the native protein, indicating that glycation has taken place. After 10 h of heating the conjugates at 90 °C, the IR bands 1634 and 1536, which belong to C=O and C–N stretching from amide I and amide II, were modified by the Maillard reaction (Wang et al., 2013). Edible insect protein incubated for 10 h has undergone a significant unfolding which causes a change in the structure of the conjugates. It is expected that the chemical changes accompanying the MR in BSFL-Glu conjugates would lead to several changes in the mid-IR spectrum as a result of depletion or consumption of some functional groups, including NH_2_, especially from lysine and the formation of others. These changes in molecular structure as influenced by the heating temperature and time are expected to cause a change in the functional properties of the resulting conjugates. The observations of this study are in agreement and further supports previous findings that FT-IR can be used as a tool to probe the structural modifications of protein-sugar systems due to Maillard conjugations (Wang et al., 2013; Jia et al., 2020; Zhang et al., 2020). Therefore, the changes in the protein structure following glycation corresponds with the results obtained from thermal stability measurements (TGA and DSC). Most importantly, this is the first study to our best knowledge to investigate the conjugation of black soldier fly larvae protein with glucose via the Maillard reaction and the analysis thereof with FT-IR, and thus provide valuable and unique information towards the ultimate adoption of conjugated edible insect proteins by the food industry as an alternative functional food ingredient with beneficial techno-functional properties.

### 3.5 Principal component analysis

Principal component analysis (PCA), a commonly used unsupervised pattern recognition chemometric tool, was performed to assess the similarities and differences between heat-treated native BSFL protein and BSFL-Glu conjugates. Furthermore, it was used to further extract the spectral changes due to the Maillard reaction as a function of reaction temperature and time using a multivariate statistical approach. In this case, PCA essentially breaks the large dataset down into orthogonal components that describe the variance in the dataset and allows one to examine only the spectral differences.

**Figure 6:**
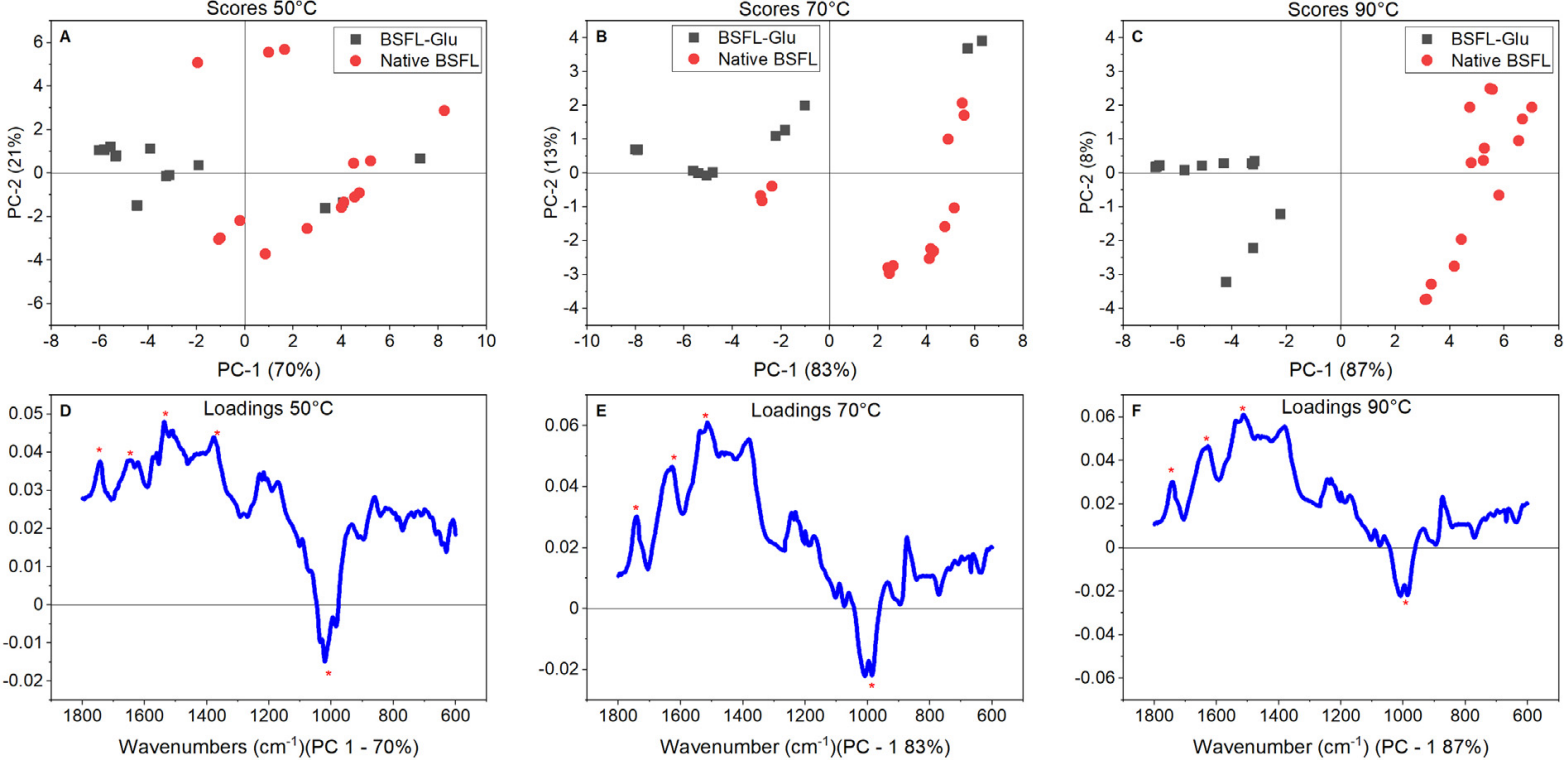
PCA score plots (A, B and C) and loadings (D, E and F) of native BSFL (red circles) and BSFL-Glu conjugates (grey squares) heated at 50, 70 and 90 °C.

Figure 6 illustrates the PCA score plots (top) and loadings plots (bottom) of native BSFL and BSFL-Glu conjugates and 90. PCA of the spectral region between proteins and sugars (1800–600 cm^−1^) was performed on the native BSFL and BSFL-Glu synthesised by heating at heated at 50, 70 and 90 °C, respectively. The score plots can be used to explain patterns and similarities of the samples, such plots show up groupings of similar spectra and consider samples to be similar if they lie closer to each other and dissimilar if they are apart from each other. PCA score plots were able to display discrimination of the native BSFL protein and BSFL-Glu conjugates. In all cases, the variance explained by the PC1 and PC2 was above 80% (Figure 6).

At 50 °C PC1 (70%) and PC2 (21%) had an accumulated total variance of 91%, at 70 °C PC1 (83%) and PC2 (13%) had an accumulated total variance of 96% and at 90 °C PC1 (87%) and PC2 (8%) had an accumulated total variance of 95% and these indicate an acceptable amount of the sample's variability explained by the respective PCAs. With reference to Figure 6 A – C, it can be observed that native BSFL protein and BSFL-Glu samples are diametrically opposed through PC1. Thus the greater distinction between the samples was achieved due to the higher reaction rate.

The loadings plot (Figure 6 D – F), show the coefficient of the linear combinations associated with each PC. In terms of the principal components, there are relationships between wavelengths and loadings. It is vital to note that variables of large loadings lie away from the origin and variables of little importance lie near the origin. The amide I, II, and carbohydrate-related bands at 1230 cm^−1^ and between 1160 and 1000 cm^−1^ are the primary absorbance peaks that contribute to sample discrimination. Table 2 displays the loadings for the first two components from PCA, indicating which of the frequencies had more influence on the discrimination of the native BSFL and BSFL-Glu conjugates. With reference to PC1, the influence was mainly due to vibrations in the amide I and amide II bands. Moreover, PC2 is influenced by the C=O stretch at 1626–1576 cm^−1^ and CH_3_ bending vibrations. This confirmed that conformational/structural changes occurred due to the Maillard reaction.

**Table 2:** Original variables (wavenumbers) from PCA with more impact on the first two principal components and the vibrational modes associated with.

Principal component	Order of relevance	Band (from – to)	Max band	Associated to
PC_1_ – 50 °C	1	1542–1521	1537	Amide II region of proteins
	2	1663–1612	1648	Amide I region of proteins
	3	1792–1701	1743	C=O stretch
	4	1399–1336	1377	Amide III region of proteins
	5	1041–1000	1018	C–O stretching vibration of carbohydrate
PC_1_ – 70 °C	1	1526–1501	1537	Amide II region of proteins
	2	1671–1699	1627	Amide I region of proteins
	3	1423–1370	1381	Amide III region of proteins
	4	1794–1706	1741	C=O stretch
	5	1021–994	1007	C–O stretching vibration of carbohydrate
PC_1_ – 90 °C	1	1670–1590	1627	Amide I region of proteins
	2	1526–1501	1513	Amide II region of proteins
	3	1774–1704	1741	C=O stretch
	4	1021–994	1006	C–O stretching vibration of carbohydrate
	5	994–979	984	C–O stretching vibration of carbohydrate
PC_2_ – 50 °C	1	1789–1704	1729	C=O stretch
	2	1626–1576	1596	N–H bend, C–N stretch
	4	1370–1341	1353	CH_3_ bending vibrations
	3	1241–1180	1217	Stretching vibration of C–O group (1228 and 1155 cm^−1^)
PC_2_ – 70 °C	1	1789–1704	1738	C=O stretch
	3	1681–1575	1591	N–H bend, C–N stretch
	2	1375–1335	1365	CH_3_ bending vibrations
	4	1241–1180	1217	Stretching vibration of C–O group (1228 and 1155 cm^−1^)
PC_2_ – 90 °C	1	1780–1703	1739	C=O stretch
	2	1393–1340	1365	CH_3_ bending vibrations
	3	1238–1194	1218	Stretching vibration of C–O group (1228 and 1155 cm^−1^)
	4	1044–978	1021	C–O stretching vibration of carbohydrate

### 3.6 SIMCA native and glycated BSFL protein classification

**Figure 7:**
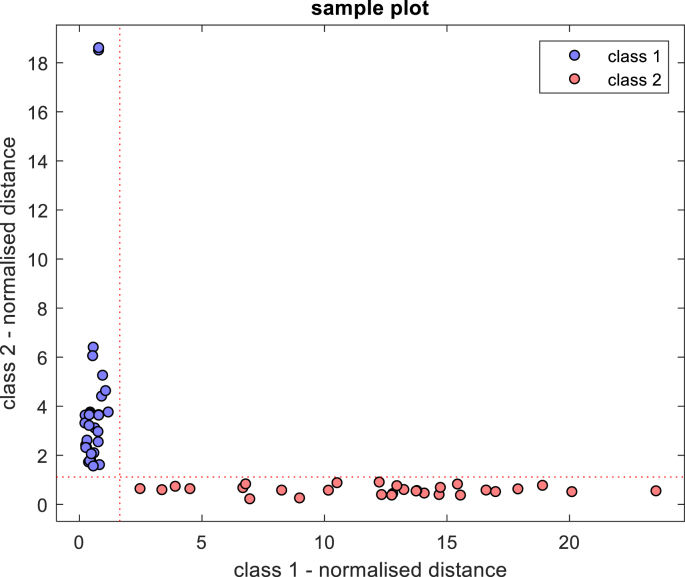
Coomans plot training step for classification of heated native BSFL protein (class 2) and BSFL-Glu conjugates (class 1) in the spectral region 1800–600 cm^−1^ (95% confidence interval).

**Figure 8:**
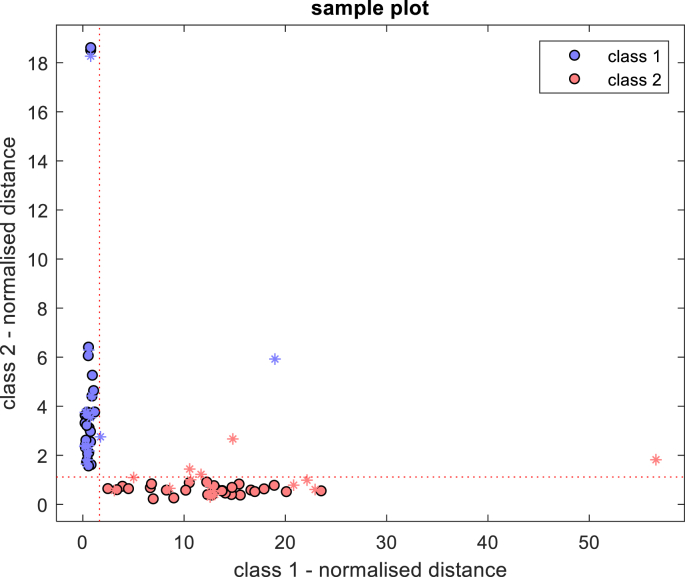
Coomans plot training and prediction step for classification of heated native BSFL protein (class 2) and BSFL-Glu conjugates (class 1) in the spectral region 1800–600 cm^−1^ (95% confidence interval).

Based on PCA, the optimal classification model for discriminating between native BSFL protein and BSFL-Glu conjugates was established using Soft independent modelling of class analogy (SIMCA), a supervised chemometric classification technique. The PCA analysis revealed that the largest differences between native BSFL protein and BSFL-Glu conjugates were due to the alterations in the amide I and amide II region 1800–1400 cm^−1^. Therefore, subsequent PCA analysis focused only on the 1800–600 cm^−1^ region. The training data set (60 samples) was able to classify native and BSFL-Glu samples and achieved 100% sample classification efficiency. This can be visualised using the Coomans' plot (Figure 7). The Coomans plot is useful to assign whether a set of unknown value samples is similar to a group of known measured samples. The model was then applied to the test (prediction) samples (30 samples). From the Coomans’ plot in Figure 8, it can be seen that there were no samples in the lower left quadrant. This indicates that in this model there were no samples that were classified as inconclusive by the model. Table 3 shows the class and global performance parameters for the prediction stage given by SIMCA. An excellent classification task was performed obtaining SEN, SPEC and PREC values of 100% for the two classes. Moreover, NER and ACC values of 100% confirm the observations given by class parameters and Coomans' plot (Figure 8). Thus, the proposed approach was able to achieve an accuracy of 91% specificity of 100% was obtained, suggesting an effective model. Therefore, the applied methodology provides a rapid and powerful tool for the successful discrimination of native and glycated BSFL samples. This study describes for the first time the application of SIMCA in heated native BSFL protein and BSFL-Glu conjugates and therefore paving way for further understanding of conjugation of edible insect protein with glucose.

**Table 3:** Class and global performance parameters from the prediction stage by SIMCA.

	Class Performance Parameters		Global Perf Parameters	
	Class 1	Class 2		
Sensitivity	100	1.00	Non error rate	1.0
Specificity	1.00	1.00	Accuracy	1.0
Precision	1.00	1.00	Error	0.0
Non assigned	0.15	0.36		

### 3.7 Microstructure analysis of native and glycated BSFL protein

**Figure 9:**
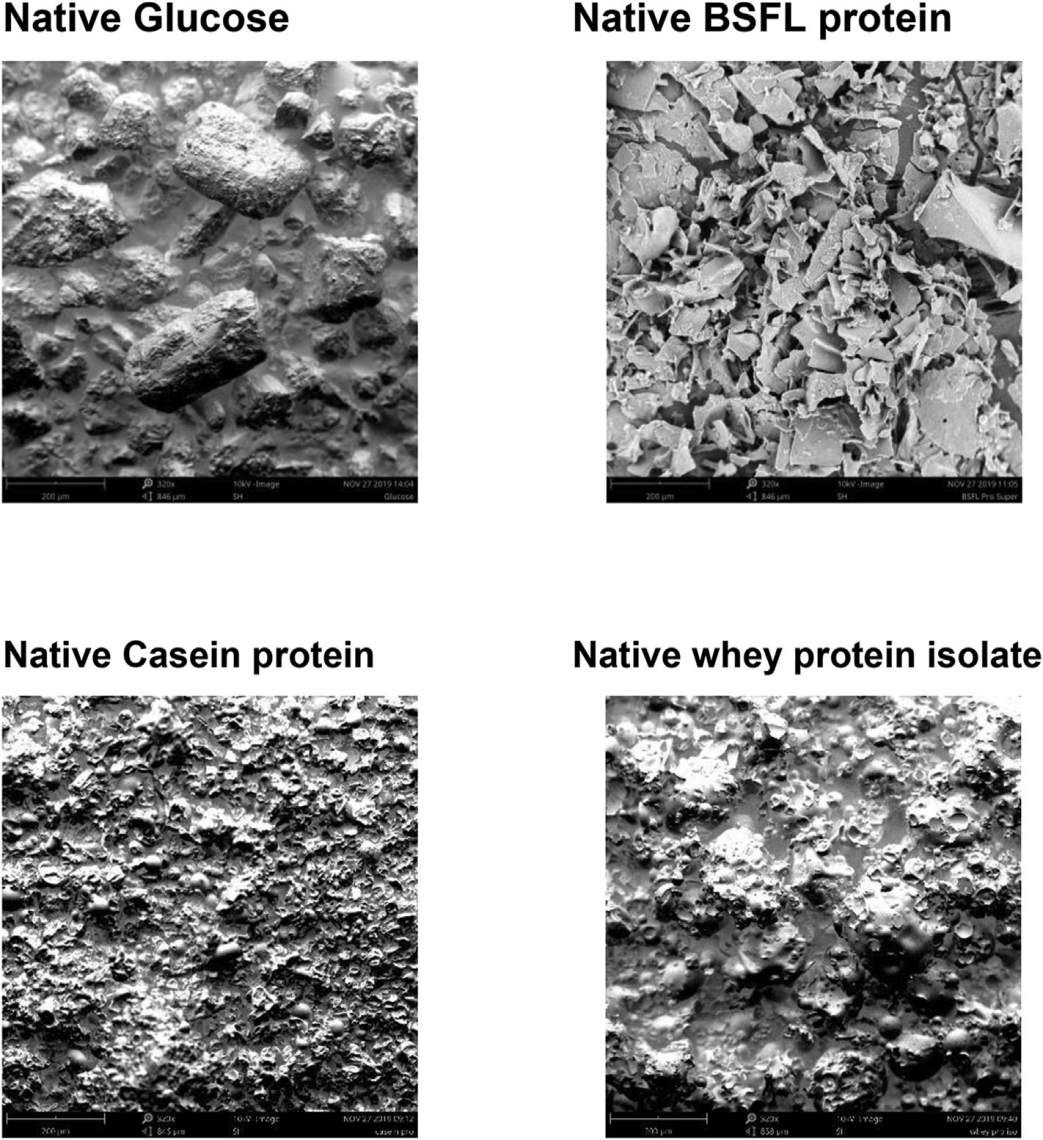
Scanning electron micrographs of native (unheated) glucose (left) and native BSFL protein (right). Images are displayed at 320 X magnification (scale bars correspond to 200 μm).

The microscopic structure of food ingredients largely influences the techno-functional properties of food systems. Hence, knowledge of the protein surface morphology is vital to understanding changes taking place in heat-treated native and conjugated BSFL protein. The surface morphology of the unheated (glucose and native BSFL protein) and heated (native BSFL protein and BSFL-Glu conjugates) samples was examined using scanning electron microscopy at 320 X magnification. In the case of unheated glucose, large particles which exhibit blocks with slightly roughened surfaces were observed (Figure 9). In contrast, native BSFL protein showed non-homogeneous flaky, thinner sheets with partially dented surfaces. The application of SEM was useful in clearly distinguishing the surface morphology of the initial reactants (glucose and BSFL protein). Casein and whey protein concentrate were used analysed with the view to compare with native BSFL. Casein exhibited a structure with wrinkled surfaces while Whey protein concentrate appeared spherical with dimpled surfaces. Both animal (dairy-based) protein structures which are commonly used in food products showed distinct surface characteristics compared to the native BSFL (Figure 9). These observations or differences in surface morphology could further be used to understand the functionalities of these proteins to develop tailor-made food ingredients with novel techno-functional properties to be incorporated in food products such as emulsions, sausages or bakery products.

**Figure 10:**
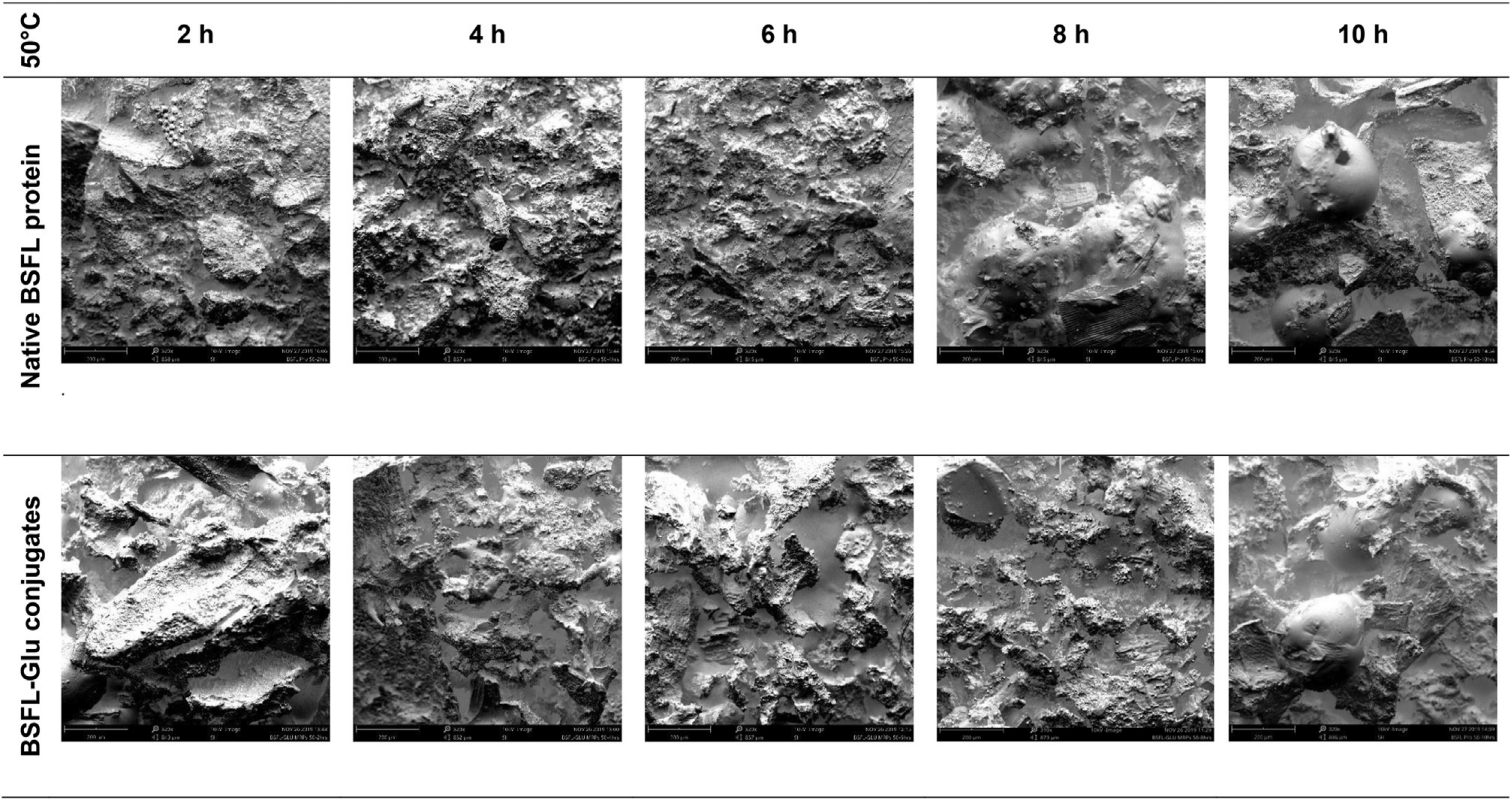
Scanning electron micrographs of heated native BSFL protein (top) and BSFL-Glu conjugates reacted at 50 °C as a function of time. Images are displayed at 320X magnification (scale bars correspond to 200 μm).

**Figure 11:**
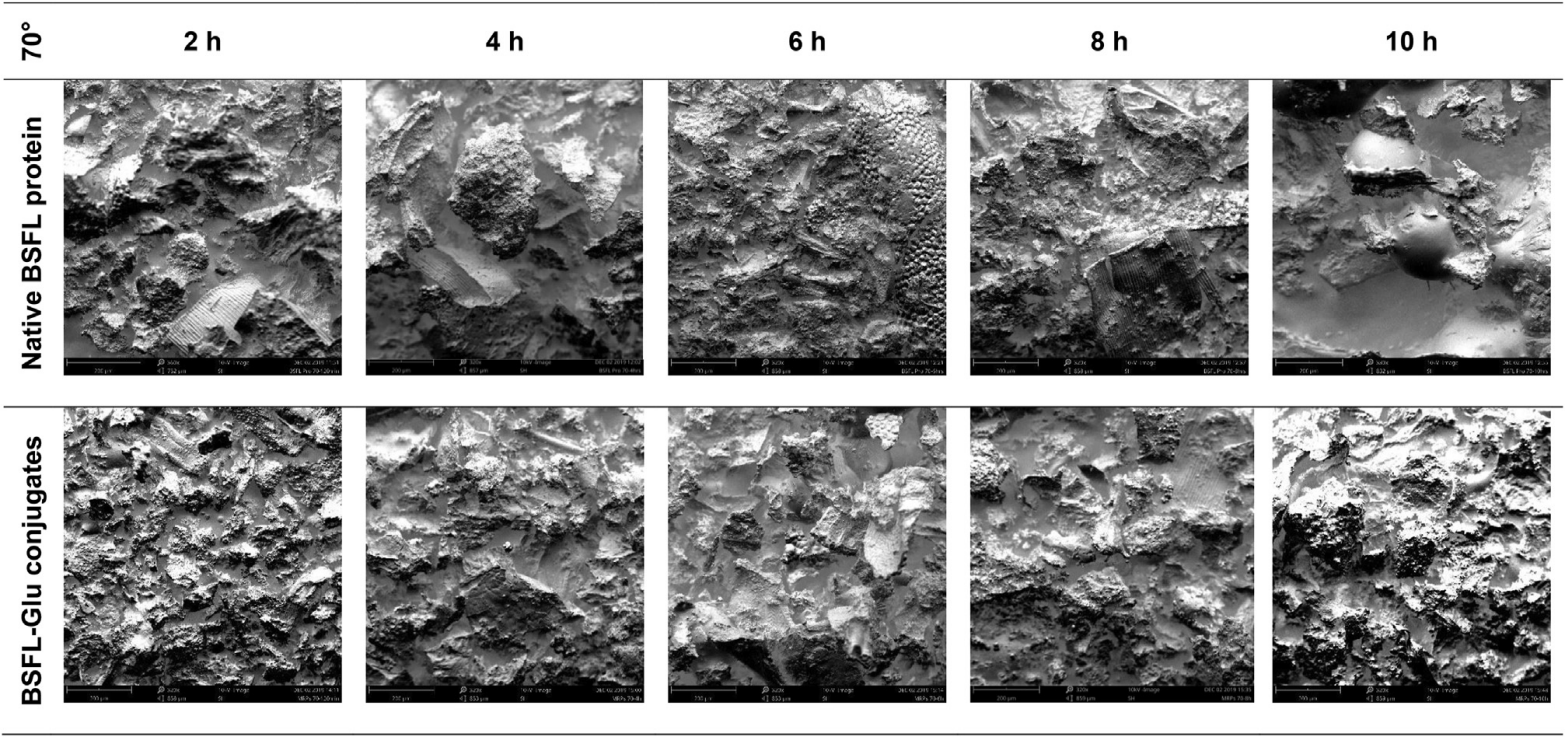
Scanning electron micrographs of heated native BSFL protein (top) and BSFL-Glu conjugates reacted at 70 °C as a function of time. Images are displayed at 320X magnification (scale bars correspond to 200 μm).

**Figure 12:**
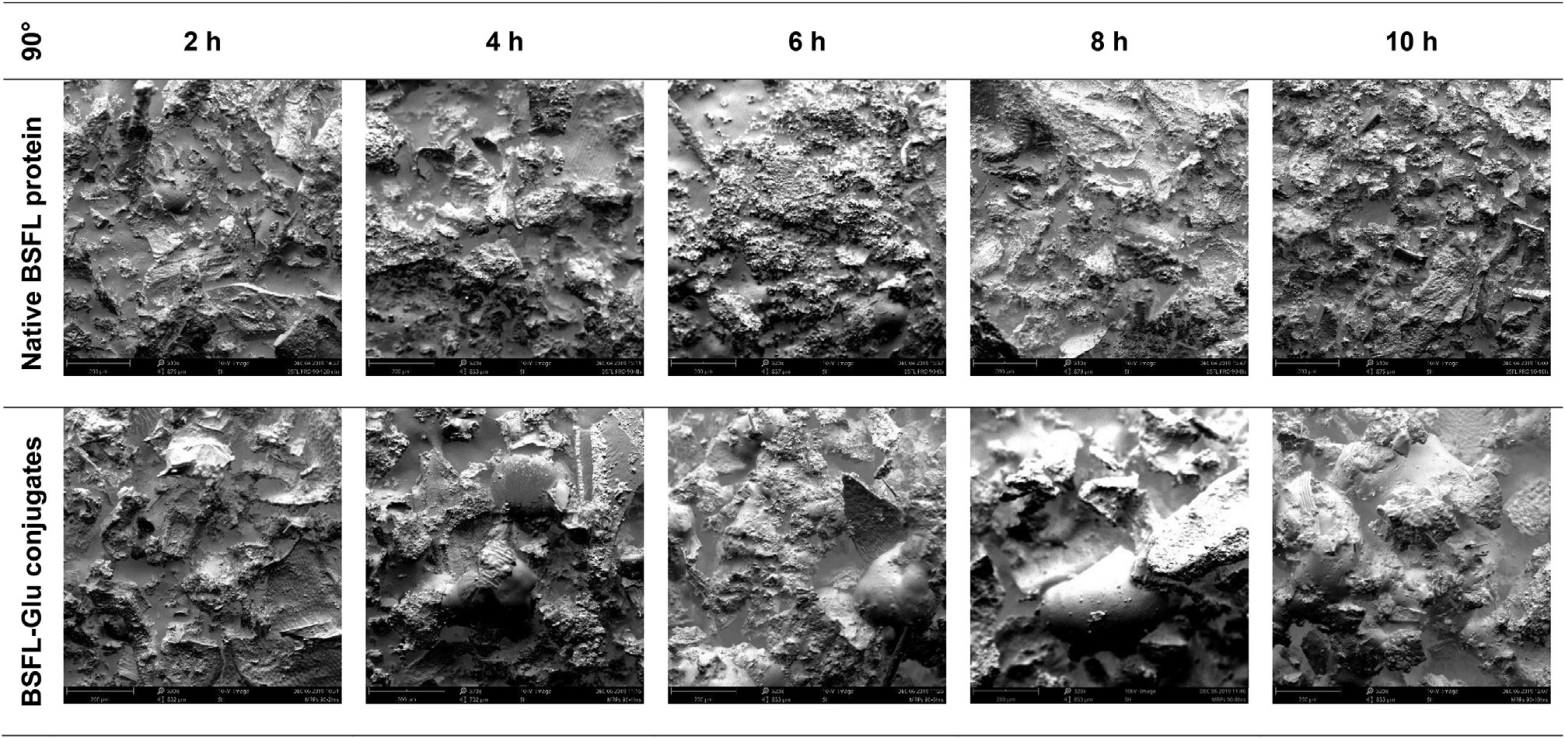
Scanning electron micrographs of heated native BSFL protein (top) and BSFL-Glu conjugates reacted at 90 °C as a function of time. Images are displayed at 320X magnification (scale bars correspond to 200 μm).

In the case of native proteins heated at 50 °C, a rough, uneven, compact and continuous surface was observed between 2 – 6 h. At longer heating times, especially at 8 and 10 h, respectively, large spherical particles were observed (Figure 10). The appearance of these structures could be attributed to the unfolding or denaturation of the native BSFL protein at prolonged heating times. BSFL-Glu conjugates had considerable roughness, less compact (more spread) compared to native BSFL protein. This loosened (less compact) surface structure with cavities is due to the wet-heat treatment which resulted in attached glucose on the surface of the BSFL protein resulting in conformational change. BSFL-Glu conjugates heated at 10 h also exhibited spherical particles similar to those observed in the native BSFL protein indicating unfolding of the structure at prolonged heating times and these results are consistent with the Zeta potential and FT-IR analyses which confirmed alteration of the protein structure through particle charge and functional groups, respectively. This phenomenon was similar to the findings reported by Wang et al. (2020) in whey protein isolate conjugated with inulin under wet-heat conditions. In general, a similar pattern was observed for samples incubated at higher reaction temperatures (Figures 11 and 12). Therefore, it can be said that the Maillard reaction results in protein unfolding and reduce the protein molecular aggregation.

## 4 Conclusions

In this study, ATR FT-IR combined with chemometric methods (PCA and SIMCA) was used to investigate the structural modification in native BSFL and BSFL-Glu conjugates. SIMCA proved to be a powerful, rapid and reliable tool for the classification of native and glycated BSFL proteins. Moreover, the apparent changes in the protein charge via the zeta potential, surface modifications revealed by the SEM and thermal properties indicate that glucose was successfully conjugated with the black soldier fly larvae protein. SEM also showed that surface structure became looser and more porous after glycation with glucose. Compared with the native BSFL protein, TGA analysis revealed that BSFL-Glu conjugates showed greater thermal stability at 90 °C. These structural modifications are vital in influencing the changes in protein techno-functionality in food applications. The techniques used were complementary and provided different levels of useful information to probe the structural modifications due to glycation. Conjugation holds a possibility for delivering novel food structures with enhanced functionalities and expand the application of BSFL-Glu conjugates in food applications. Further work is required to better understand the impact of glycation of edible insect-derived proteins on the loss of nutritive value of the proteins involved.

## Declarations

### Author contribution statement

Vusi Vincent Mshayisa: Conceived and designed the experiments; Performed the experiments; Analyzed and interpreted the data; Wrote the paper.

Jessy Van Wyk: Analyzed and interpreted the data; Contributed reagents, materials, analysis tools or data.

Bongisiwe Zozo: Performed the experiments.

Silvio D. Rodríguez: Analyzed and interpreted the data.

### Funding statement

This research did not receive any specific grant from funding agencies in the public, commercial, or not-for-profit sectors.

### Data availability statement

Data will be made available on request.

### Declaration of interests statement

The authors declare no conflict of interest.

### Additional information

No additional information is available for this paper.

## References

[bib1] Balan B., Dhaulaniya A.S., Jamwal R., Sodhi K.K., Kelly S., Cannavan A., Singh D.K. (2020). Vibrational Spectroscopy Application of Attenuated Total Reflectance-Fourier Transform Infrared (ATR-FTIR) Spectroscopy Coupled with Chemometrics for Detection and Quantification of Formalin in Cow Milk.

[bib2] Boland M.J., Rae A.N., Vereijken J.M., Meuwissen M.P.M., Fischer A.R.H., Van-Boekel M.A.J.S., Rutherfurd S.M., Gruppen H., Moughan P.J., Hendriks W.H. (2013). The future supply of animal-derived protein for human consumption. Trends Food Sci. Technol..

[bib3] Chevalier F., Chobert J.M., Popineau Y., Nicolas M.G., Haertlé T. (2001). Improvement of functional properties of β-lactoglobulin glycated through the Maillard reaction is related to the nature of the sugar. Int. Dairy J..

[bib4] Corzo-Martínez M., Moreno F.J., Villamiel M., Rodríguez Patino J.M., Carrera Sánchez C. (2017). Effect of glycation and limited hydrolysis on interfacial and foaming properties of bovine β-lactoglobulin. Food Hydrocoll..

[bib5] Gould J., Wolf B. (2018). Interfacial and emulsifying properties of mealworm protein at the oil/water interface. Food Hydrocoll..

[bib6] Huang C., Feng W., Xiong J., Wang T., Wang W., Wang C., Yang F. (2018). Impact of drying method on the nutritional value of the edible insect protein from black soldier fly (*Hermetia illucens L.*) larvae: amino acid composition, nutritional value evaluation, in vitro digestibility, and thermal properties. Eur. Food Res. Technol..

[bib7] Jia C., Cao D., Ji S., Lin W., Zhang X., Muhoza B. (2020). Whey protein isolate conjugated with xylo-oligosaccharides via maillard reaction: characterization, antioxidant capacity, and application for lycopene microencapsulation. LWT - Food Sci. Technol. (Lebensmittel-Wissenschaft -Technol.).

[bib8] Kim H.-W., Setyabrata D., Lee Y.J., Jones O.G., Kim Y.H.B. (2016). Pre-treated mealworm larvae and silkworm pupae as a novel protein ingredient in emulsion sausages. Innovat. Food Sci. Emerg. Technol..

[bib9] Ladjal-Ettoumi Y., Boudries H., Chibane M., Romero A. (2016). Pea, chickpea and lentil protein isolates: physicochemical characterization and emulsifying properties. Food Biophys..

[bib10] Leni G., Caligiani A., Sforza S. (2019). Killing method affects the browning and the quality of the protein fraction of Black Soldier Fly (*Hermetia illucens*) prepupae: a metabolomics and proteomic insight. Food Res. Int..

[bib11] Liu J., Ru Q., Ding Y. (2012). Glycation a promising method for food protein modification: physicochemical properties and structure, a review. Food Res. Int..

[bib12] Medrano A., Abirached C., Panizzolo L., Moyna P., Añón M.C. (2009). The effect of glycation on foam and structural properties of β-lactoglobulin. Food Chem..

[bib13] Mellado-Carretero J., Kaade W., Ferrando M., Güell C., Lamo-Castellví S. (2019). Attenuated total reflectance Fourier transform midinfrared spectroscopy combined with multivariate analysis, a novel approach to monitor maillard reaction. J. Food Sci..

[bib14] Mshayisa V.V. (2016). Antioxidant effects of Maillard reaction products (MRPs) derived from glucose-casein model systems (MTech thesis).

[bib15] Mshayisa V.V., Van Wyk J. (2021). Hermetia illucens protein conjugated with glucose via maillard reaction: antioxidant and techno-functional properties. Int. J. Food Sci..

[bib16] O’Mahony J.A., Drapala K.P., Mulcahy E.M., Mulvihill D.M. (2017). Controlled glycation of milk proteins and peptides: functional properties. Int. Dairy J..

[bib17] Oliver C.M., Kher A., McNaughton D., Augustin M.A. (2009). Use of FTIR and mass spectrometry for characterization of glycated caseins. J. Dairy Res..

[bib18] Patel S., Ansar H., Suleria R., Rauf A. (2019). Accepted Manuscript Edible insects as innovative foods: nutritional and functional assessments. Trends Food Sci. Technol..

[bib19] Pirestani S., Nasirpour A., Keramat J., Desobry S., Jasniewski J. (2018). Structural properties of canola protein isolate-gum Arabic Maillard conjugate in an aqueous model system. Food Hydrocoll..

[bib20] Rodríguez S.D., Rolandelli G., Buera M.P. (2019). Detection of quinoa fl our adulteration by means of FT-MIR spectroscopy combined with chemometric methods. Food Chem..

[bib21] Schwenzfeier A., Helbig A., Wierenga P.A., Gruppen H. (2013). Emulsion properties of algae soluble protein isolate from *Tetraselmis sp*. Food Hydrocoll..

[bib22] Tang C.H., Sun X., Foegeding E.A. (2011). Modulation of physicochemical and conformational properties of kidney bean vicilin (phaseolin) by glycation with glucose: implications for structure-function relationships of legume vicilins. J. Agric. Food Chem..

[bib23] Van-der Spiegel M., Noordam M.Y., van der Fels-Klerx H.J. (2013). Safety of novel protein sources (insects, microalgae, seaweed, duckweed, and rapeseed) and legislative aspects for their application in food and feed production. Compr. Rev. Food Sci. Food Saf..

[bib24] Wang W.D., Li C., Bin Z., Huang Q., You L.J., Chen C., Fu X., Liu R.H. (2020). Physicochemical properties and bioactivity of whey protein isolate-inulin conjugates obtained by Maillard reaction. Int. J. Biol. Macromol..

[bib25] Wang W.Q., Bao Y.H., Chen Y., Wen-qiong W., Yi-hong B., Ying C. (2013). Characteristics and antioxidant activity of water-soluble Maillard reaction products from interactions in a whey protein isolate and sugars system. Food Chem..

[bib26] Wang Y.-S., Shelomi M. (2017). Review of black soldier fly (*Hermetia illucens*) as animal feed and human food. Foods.

[bib27] Zhang X., Li X., Liu L., Wang L., Fanny A., Bora M., Du L. (2020). Covalent conjugation of whey protein isolate hydrolysates and galactose through Maillard reaction to improve the functional properties and antioxidant activity. Int. Dairy J..

